# Supplementary material for: Patterns of Cerebrospinal Fluid Alzheimer’s Dementia Biomarkers in People Living with HIV: Cross-Sectional Study on Associated Factors According to Viral Control, Neurological Confounders and Neurocognition
Source: Viruses. 2022 Apr 4;14(4):753. doi: 10.3390/v14040753 (PMC9031633; doi:10.3390/v14040753)

Supplementary Material

Supplementary Table S 1. Univariate and Multivariate binary logistic regression for demographic, clinical and viro-immunological factors associated with CSF patterns of Alzheimer's Dementia biomarkers in patients with undetectable viremia

Parameter	Univariate		Multivariate	
	CSF pattern B	CSF pattern C	CSF pattern B	CSF pattern C
	OR (95%CI), p-value		aOR (95%CI), p-value	
**Caucasian, n**	2.50 (0.27-23.36), .422	0 (0-0), .999	-	-
**Age, years**	***1.07 (1.002-1.15), .048***	1.02 (0.92-1.12), .695	-	-
**Male sex, n**	0.78 (0.18-3.40), .736	0.45 (0.061-3.33), .436	-	-
**Exposure risk, n**			-	-
**MSM**	Ref.	Ref.		
**Heterosexual**	3.33 (0.74-15.08), .118	0 (0-0), .997		
**Previous IDU**	0.33 (0.033-3.25), .340	0 (0-0), .997		
**HCV co-infection, n**	0.72 (0.13-3.86), .699	1.83 (0.25-13.49), .552	-	-
**HBV co-infection, n**	0.0 (0.0-0.0), .998	0 (0-0), .998	-	-
**Plasma HIV-RNA, Log10 cp/mL**	1.32 (0.44-3.94), .615	1.99 (0.34-11.48), .444	-	-
**CSF HIV-RNA, Log10 cp/mL**	0.94 (0.41-2.14), .887	3.22 (0.85-12.19), .086	-	1.61 (0.28-9.23), .591
**CSF HIV-RNA <50 cp/mL, n**	1.57 (0.19-13.24), .679	0.20 (0.026-1.47), .114	-	-
**CSF escape, n**	0.51 (0.61-4.28), .535	4.08 (0.55-30.48), .170	-	-
**Current CD4+ T-cells, cells/mmc**	1.001 (0.99-1.003), .590	1.002 (0.99-1.005), .312	-	-
**CD4+ count nadir, cells/mmc**	1.001 (0.99-1.006), .618	0.99 (0.98-1.007), .467	-	-
**HIV infection, months**	1.003 (0.99-1.01), .449	***1.017 (1.003-1.032), .020***	-	1.007 (0.99-1.03), .431
**Time on current cART, months**	0.99 (0.95-1.03), .620	0.99 (0.96-1.04), .975	-	-
**Time of suppression, months**	1.003 (0.99-1.02), .653	1.00 (0.98-1.019), .964	-	-
**cART regimens, n**			-	-
**Dual**	Ref	Ref.		
**PI-NRTI**	0.28 (0.024-3.29), .313	0.37 (0.033-3.38), .414		
**INI-NRTI**	0.88 (0.11-6.78), .902	0.62 (0.084-6.52), .784		
**nNRTI-NRTI**	1.47 (0.19-11.59), .716	0.86 (0.09-10.00), .852		
**More than 3 drugs**	1.10 (0.14-8.56), .927	1.07 (0.11-7.93), .947		
**Clinical diagnosis, n**			-	-
**N/N issues**	0.63 (0.063-6.17), .687	Omitted*		
**Symptomatic NCI**	Omitted*	Omitted*		
**Asymptomatic**	Ref.	Ref.		
**Isolated brain MRIa**	0.26 (0.047-1.36), .127	0.66 (0.39-11.01), .771		
**HIV-related syndromes**	0.21 (0.023-1.92), .166	2.08 (0.18-24.51), .559		
**CSF ptau, pg/mL**	0.97 (0.92-1.03), .354	***1.09 (1.02-1.17), .011***	-	1.06 (0.99-1.13), .115
**CSF Neopterin, ng/mL**	0.89 (0.36-2.21), .789	0.46 (0.068-3.16), .432	-	-
**CSF S100b, pg/mL**	0.99 (0.99-1.006), .417	1.00 (0.99-1.006), .573	-	-
**CSAR**	0.74 (0.51-1.09), .125	0.72 (0.40-1.3), .279	-	-
**Tourtellote index**	(0.85-1.06), .351	0.98 (0.90-1.08), .750	-	-
**CSF IgG synthesis, %**	0.99 (0.95-1.03), .526	1.005 (0.96-1.05), .823	-	-
**CSF cells, cells/mmc**	0.88 (0.37-2.11), .797	***1.84 (1.07-3.18), .028***	-	1.43 (0.64-3.19), .383
**CSF proteins, mg/dL**	0.97 (0.91-1.02), .247	0.99 (0.92-1.05), .660	-	-
**CSF glucose, mg/dL**	1.03 (0.96-1.09), .414	0.94 (0.83-1.06), .299	-	-

Supplementary Table S 2. Univariate and Multivariate binary logistic regression for demographic, clinical and viro-immunological factors associated with CSF patterns of Alzheimer's Dementia biomarkers in patients with detectable viremia

**Parameter**	**Univariate**		**Multivariate**	
	**CSF pattern B**	**CSF pattern C**	**CSF pattern B**	**CSF pattern C**
	**OR (95%CI), p-value**		**aOR (95%CI), p-value**	
**Caucasian, n**	2.35 (0.54-10.35), .258	0.78 (0.16-3.83), .764	-	-
**Age, years**	***1.13 (1.05-1.22), .001***	1.02 (0.97-1.08), .367	***1.16 (1.03-1.31), .017***	-
**Male sex, n**	0.37 (0.093-1.49), .163	0.42 (0.12-1.43), .165	-	-
**Exposure risk, n**			-	-
**MSM**	Ref.	Ref.		
**Heterosexual**	1.33 (0.28-6.34), .718	0.67 (0.19-2.33), .533		
**Previous IDU**	0.86 (0.13-5.47), .873	0.32 (0.063-1.69), .182		
**HCV co-infection, n**	0.45 (0.054-3.79), .464	2.29 (0.61-8.62), .222	-	-
**HBV co-infection, n**	0.78 (0.16-3.82), .763	0 (0-0), .998	-	-
**Plasma HIV-RNA, Log10 cp/mL**	0.79 (0.52-1.23), .299	0.88 (0.60-1.29), .515	-	-
**CSF HIV-RNA, Log10 cp/mL**	1.38 (0.80-2.39), .243	1.56 (0.99-2.47), .057	-	0.71 (0.27-1.86), .486
**CSF HIV-RNA <50 cp/mL, n**	0.88 (0.10-7.72), .912	0.20 (0.011-3.62), .278	-	-
**CSF escape, n**	0.99 (0.99-1.001), .999	1.08 (0.93-1.24), .126	-	-
**Current (nadir) CD4+ T-cells, cells/mmc**	***1.004 (1.001-1.006), .009***	0.99 (0.99-1.003), .458	1.002 (0.99-1.008), .390	-
**On cART, n**	1.61 (0.37-6.95), .524	0.89 (0.22-3.42), .851	-	-
**HIV infection, months**	1.003 (0.99-1.009), .199	1.003 (0.99-1.008), .263	-	-
**Clinical diagnosis, n**			-	-
**N/N issues**	2.22 (0.18-27.26), .532	0.87 (0.089-8.56), .909		
**Symptomatic NCI**	Omitted*	Omitted*		
**Asymptomatic**	Ref.	Ref.		
**Isolated brain MRIa**	1.11 (0.15-8.31), .918	0.64 (0.14-2.88), .557		
**HIV-related syndromes**	4.70 (0.78-28.17), .114	2.92 (0.72-11.85), .135		
**CSF ptau, pg/mL**	***0.88 (0.79-0.98), .021***	***1.08 (1.04-1.13), <.0005***	//	//
**CSF Neopterin, ng/mL**	***1.24 (1.06-1.44), .005***	***1.34 (1.13-1.58), .001***	1.19 (0.90-1.58), .214	***1.47 (1.10-1.96), .009***
**CSF S100b, pg/mL**	1.00 (0.99-1.004), .853	1.00 (0.99-1.004), .823	-	-
**CSAR**	1.11 (0.93-1.33), .232	1.11 (0.97-1.27), .135	-	-
**Tourtellote index**	***1.03 (1.01-1.05), .004***	***1.02 (1.004-1.04), .015***	1.03 (0.97-1.09), .299	1.02 (0.97-1.06), .428
**CSF IgG synthesis, %**	1.02 (0.99-1.044), .254	1.004 (0.98-1.03), .787	-	-
**CSF cells, cells/mmc**	1.02 (0.98-1.07), .359	***1.04 (1.006-1.07), .020***	-	0.93 (0.79-1.09), .363
**CSF proteins, mg/dL**	1.03 (0.99-1.05), .062	***1.04 (1.02-1.07), .002***	0.91 (0.82-1.02), .101	1.007 (0.95-1.07), .814
**CSF glucose, mg/dL**	***0.89 (0.81-0.97), .012***	0.93 (0.86-1.01), .075	***0.85 (0.75-0.97), .019***	1.01 (0.92-1.11), .823

*No cases.

Supplementary Table S 3. Univariate and Multivariate binary logistic regression for demographic, clinical and viro-immunological factors associated with CSF patterns of Alzheimer's Dementia biomarkers in patients with central nervous system confounding conditions

Parameter	Univariate		Multivariate	
	CSF pattern B	CSF pattern C	CSF pattern B	CSF pattern C
	OR (95%CI), p-value		aOR (95%CI), p-value	
**Caucasian, n**	***0.17 (0.029-0.99), .050***	2.79 (0.61-12.67), .185	13.97 (0.59-326.53), .101	-
**Age, years**	0.97 (0.91-1.03), .358	***0.91 (0.85-0.97), .007***	-	0.91 (0.82-1.005), .062
**Male sex, n**	***0.12 (0.023-0.60), .010***	0.51 (0.14-1.87), .308	0.28 (0.028-2.88), .286	-
**Exposure risk, n**				-
**MSM**	Ref.	Ref.	Ref.	
**Heterosexual**	4.85 (0.46-51.66), .191	1.41 (0.41-4.82), .581	0.58 (0.015-22.28), .580	
**Previous IDU**	9.33 (0.91-95.57), .060	0.87 (0.19-4.08), .865	7.59 (0.46-123.91), .155	
**HCV co-infection, n**	0-0	0.69 (0.13-3.69), .660	-	-
**HBV co-infection, n**	3.42 (0.27-43.70), .345	4.39 (0.66-29.06), .125	-	-
**Plasma HIV-RNA, Log10 cp/mL**	0.84 (0.57-1.22), .357	1.22 (0.94-1.59), .138	-	-
**CSF HIV-RNA, Log10 cp/mL**	0.92 (0.58-1.48), .745	1.25 (0.91-1.72), .164	-	-
**CSF HIV-RNA <50 cp/mL, n**	1.38 (0.29-6.67), .685	0.89 (0.26-3.01), .848	-	-
**CSF escape, n**	0.97 (0.17-5.53), .972	1.12 (0.32-3.86), .859	-	-
**Current CD4+ T-cells, cells/mmc**	1.001 (0.99-1.003), .602	0.99 (0.99-1.001), .351	-	-
**Nadir CD4+ T-cells, cells/mmc**	0.99 (0.99-1.005), .538	0.99 (0.99-1.003), .385	-	-
**HIV infection, months**	0.99 (0.99-1.006), .771	0.99 (0.99-1.001), .099	-	0.99 (0.99-1.007), .651
**On cART, n**	1.78 (0.32-9.88), .511	0.93 (0.30-2.89), .902	-	-
**Clinical diagnosis, n**			-	-
**CNS Infections**	1.20 (0.25-5.68), .818	1.87 (0.57-6.19), .304		
**Others**	Ref.	Ref.		
**CSF ptau, pg/mL**	***0.80 (0.68-0.94), .010***	***1.05 (1.02-1.09), .005***	//	//
**CSF Neopterin, ng/mL**	1.02 (0.87-1.19), .813	1.12 (0.98-1.28), .085	-	1.03 (0.89-1.19), .651
**CSF S100b, pg/mL**	1.00 (0.99-1.004), .832	1.002 (1.00-1.004), .104	-	-
**CSAR**	1.12 (0.99-1.27), .078	0.98 (0.85-1.13), .798	1.10 (0.93-1.30), .256	-
**Tourtellote index**	1.01 (0.99-1.02), .118	1.01 (0.99-1.02), .136	-	-
**CSF IgG synthesis, %**	1.005 (0.96-1.06), .843	***1.04 (1.007-1.07), .017***	-	1.04 (0.99-1.08), .073
**CSF cells, cells/mmc**	1.02 (0.99-1.04), .113	1.005 (0.99-1.02), .418	-	-
**CSF proteins, mg/dL**	1.01 (0.99-1.03), .123	1.01 (0.99-1.02), .401	-	-
**CSF glucose, mg/dL**	1.01 (0.97-1.06), .622	0.98 (0.95-1.03), .596	-	-

Supplementary Figure S1. CSF AD biomarkers correlations with age in HIV-positive clinical groups and HIV-negative subjects with AD.

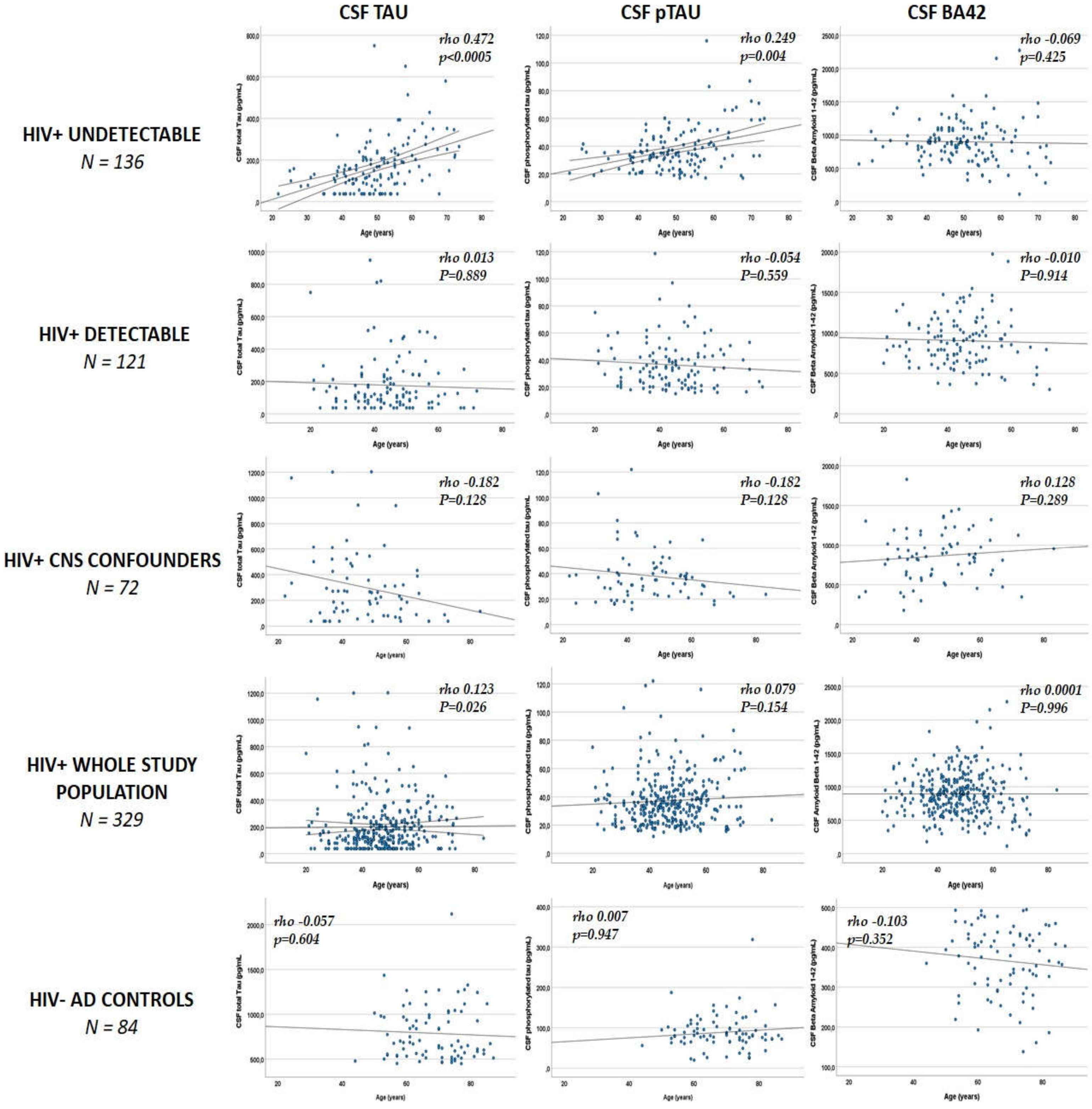

Supplement: Supplementary file 1 [file viruses-14-00753-s001.zip › viruses-1656907-supplementary.pdf]
